# Next-generation T cell engagers for cancer and autoimmune diseases

**DOI:** 10.3389/fimmu.2026.1885909

**Published:** 2026-07-22

**Authors:** Jennifer S. Michaelson, Catherine C. Henry, Karsten Sauer, Patrick A. Baeuerle

**Affiliations:** 1 Cullinan Therapeutics, Inc., Cambridge, MA, United States; 2 Department of Immunology and Microbiology, The Scripps Research Institute, La Jolla, CA, United States; 3 Institute for Immunology, Biomedical Center, Ludwig Maximilian University of Munich, Martinsried-Planegg, Germany

**Keywords:** autoimmune disease, B cell depletion, bispecific antibody, conditional, costimulation, logic-gated, T cell, T cell engager (TCE)

## Abstract

T cell-engaging antibody constructs (TCEs) have emerged as a potent modality to treat cancer and autoimmune diseases. Twelve TCEs have been approved by the FDA and EMA for the treatment of hematological malignancies or solid tumors. Despite the varying designs and binding properties, they all lead to robust single-agent efficacy and approvals in refractory or relapsed leukemia, lymphomas, multiple myeloma (MM), small cell lung cancer, EpCAM-expressing cancers or uveal melanoma. Where comparisons can be deduced, TCEs appear to achieve response rates like those obtained with CAR-T cell therapies. Given the success of the first generation of TCEs, considerable attempts are underway to further improve upon this modality. With the goal of expanding TCEs into other malignant and autoimmune indications, and to further enhance efficacy and improve safety, a multitude of novel TCEs are currently in preclinical and clinical development. Here we review the current approaches to developing next-generation TCEs that can widen the therapeutic index, address heterogeneous target expression, and thereby potentially improve efficacy and safety.

## Introduction

**Figure 1:**
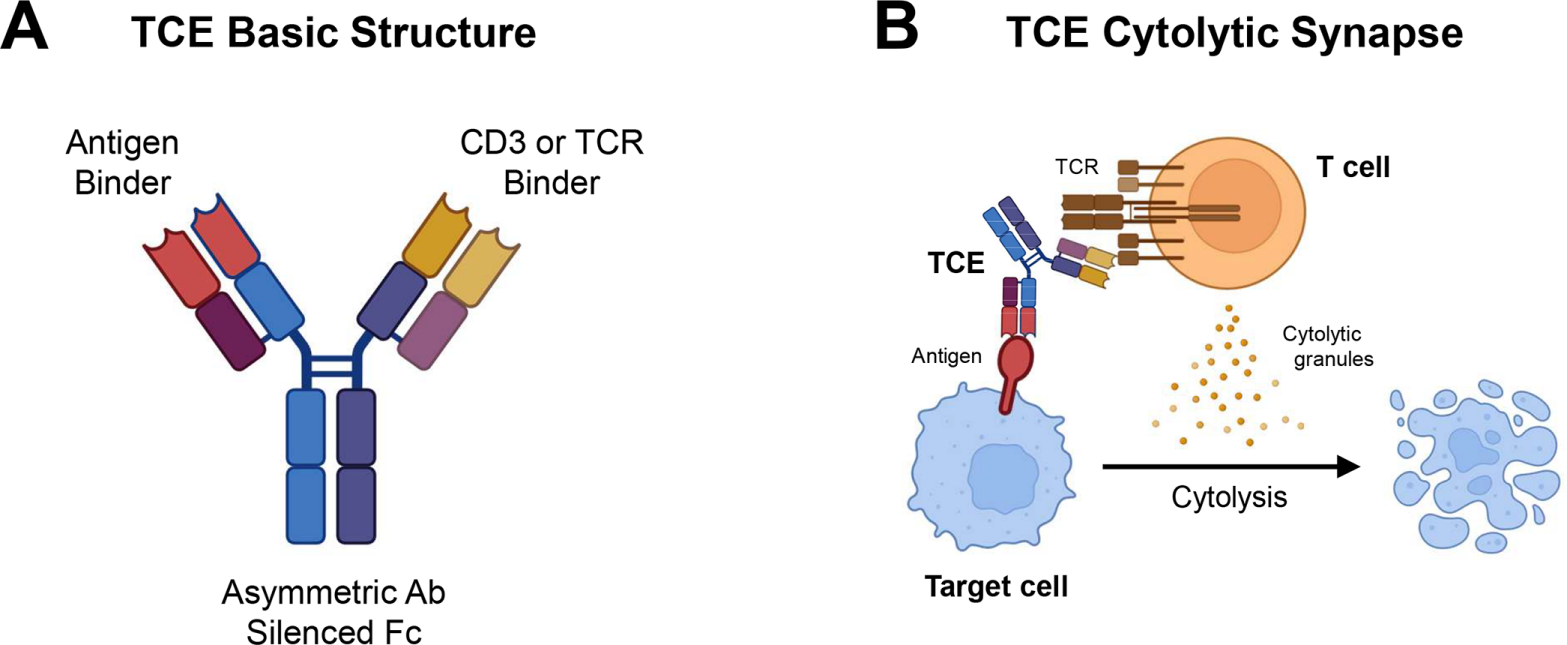
TCE basic structure and mechanism of action. **(A)** The basic structure of a TCE contains at least one antibody-derived binding domain, also termed “binder”, each for a targeted antigen (red) and for a component of the TCR complex, typically CD3ϵ (golden). Most TCEs also contain half-life extending domains such as the immunoglobulin G (IgG) Fc-region shown here. To avoid engagement of innate mechanisms which could deplete the engaged T cells or reduce TCE half-life, Fc-regions usually carry silencing mutations. A common TCE format is that of an asymmetric IgG1 where disulfide bonds link two different heavy and light chains (blue, purple). **(B)** By binding to both an antigen on a target cell and the TCR on a T cell, TCEs lead to the formation of a cytolytic or immune synapse. This activates the T cell to secrete cytolytic granules and kill the target cell via cytolysis (1). Created partly in BioRender. Sauer, K (2026). https://BioRender.com/b0yl9kw.

T cell-engaging antibody constructs (TCEs; **Figure 1A**) represent a unique approach to engage all cytotoxic T lymphocytes in the body for redirected lysis of cancer or autoimmune cells. This is possible by binding the invariant CD3ϵ chain of the T cell receptor complex (TCR) with one arm of the TCE, and a select surface antigen with the other arm (**Figure 1B**). This enables T cell activation and subsequent T cell-mediated cellular cytotoxicity (TDCC) regardless of the TCR specificity of the T cell or expression of peptide/MHC complexes on the target cells. Bypassing the TCR/peptide/MHC interaction allows TCEs to access a large variety of cell surface targets, to theoretically engage all T cell phenotypes, and to eliminate cancer cells that have lost peptide/MHC expression as an ultimate mechanism of immune evasion (1).

Connecting CD3ϵ with a cell surface target antigen for target cell lysis is possible by a wide variety of antibody-based TCE constructs and surface targets that ultimately all trigger formation of a cytolytic synapse between T cells and target cells. Thus far, seven target antigens have been used for commercial TCEs and more are targeted by TCEs in advanced clinical trials (1–3). Binding affinities of TCEs for CD3ϵ and target antigens can greatly vary and so can dose levels used for therapy. Of note, all approved TCEs only provide T cells with signal 1 (i.e., TCR activation), which alone may lead to exhaustion and activation-induced T cell death (AICD). This impediment may be balanced by the excess of non-exhausted peripheral and tissue-resident T cells available for engagement by the TCE modality. Combinations with costimulatory agonists (1) or next-generation costimulatory TCE designs providing ‘signal 2’, as discussed in this review, may offer additional solutions to counter T cell exhaustion.

The adverse events of TCEs fall into two general categories. One results from the activation of T cells upon encounter of target cells and formation of a cytolytic synapse and is shared with other T cell-engaging therapeutics such as CAR-T cells. T cell activation leads to the release of pro-inflammatory cytokines and chemokines and the induction of further cytokines in stimulated immune cells such as macrophages (1). Key cytokines involved in what can escalate into cytokine release syndrome (CRS) are TNF-α, γ-interferon and IL-6. The neutralization of IL-6 by the approved monoclonal antibody tocilizumab is a powerful means to treat CRS, but likewise steroids such as dexamethasone or prednisolone and subcutaneous administration of TCEs can mitigate first-dose cytokine-related adverse events (4). Cytokine release is typically observed early upon the start of TCE treatment. The initial TCE treatments often cause tachyphylaxis, a rapid decrease in response to drug following administration, thus subsequent TCE treatments typically no longer result in significant cytokine release. Most commercial TCEs rarely elicit CRS exceeding grade 2. Another, but less frequent, side effect of T cell activation is immune effector cell-associated neurotoxicity syndrome (ICANS) (4).

The other category of adverse events stems from the choice of target antigen. While antibody-based therapies, like monoclonal antibodies (mAbs) and antibody-drug conjugates (ADCs), can take advantage of differential expression of the target antigen between diseased and normal tissues to achieve a therapeutic window, TCEs require exquisitely tumor-specific expression or accessibility of target antigens. TCEs typically require only a low, often two-digit, copy number of target antigens for target cell lysis, akin to the low copy number of peptide/MHC complexes recognized by specific T cell clones (1, 5). Consequently, even if there is low expression of target antigen on critical organs, cytotoxic T cells can damage healthy tissues and eventually mediate serious side effects. This was demonstrated for TCEs recognizing EGFR and HER2 in non-human primate studies (6) and led to the discontinuation of many other TCE programs at the preclinical stage or in early clinical trials (7). Next-generation logic-gated TCEs, as discussed in this review, potentially enable the use of target antigens with some degree of off-tumor expression.

The response rates (RR) and survival impact of TCEs vary with the tumor load, state of disease, indication, and target and epitope selection. A higher tumor burden is typically associated with a lower RR and higher severity of side effects. This was apparent with the first approved TCE, blinatumomab, in patients with acute B lymphoblastic leukemia (ALL). While the TCE showed an 81% complete response (CR) rate and low side effects in patients with minimal residual disease (MRD), the CR rate was only 34% in relapsed/refractory ALL, and CRS and ICANS of grade 3 and higher were seen (8–10). In solid tumor indications, RRs with TCEs are typically lower than in blood cancers, more in the range of 20-40%, and there still is limited experience in the setting of combination therapies (1, 11). This difference in RRs may be attributed to the accessibility of target cells, which is greater in blood and lymphoid organs compared to tumor tissue, or due to the immunosuppressive tumor microenvironment (TME) that may limit T cell performance and migration into solid tumors. Moreover, hematological malignancies offer lineage-specific target antigens and often can afford ablation of the healthy blood compartment, which can be reconstituted from the bone marrow (12, 13). Very high RRs can be achieved by the combination of TCEs with standard of care, such as chemotherapy, mAbs, ADCs, tyrosine kinase inhibitors, checkpoint inhibitors or IMiDs (immunomodulatory imide drugs), which can all augment RRs in lymphoma patients (8, 14, 15).

A deep and comprehensive depletion of autoreactive B cells alongside the normal B cell compartment followed by reconstitution of a healthy B cell repertoire from progenitors, also referred to as an ‘immune reset’, is the goal of many TCEs in current clinical development for a diverse range of autoimmune diseases (16). This appears easier than eradicating the larger load of malignant B cells in leukemia and lymphoma lesions. However, whether a full reset with complete B cell depletion/repletion in tissues resulting in long-term remission or cures can be achieved is still under investigation. Wrapping up, it is evident that TCEs offer room for improvement regarding both efficacy and safety. Moreover, there is an ongoing need for additional target antigens, which cannot be simply borrowed from approved ADCs or mAbs, to enable expansion into more indications. The opportunity for broadening the indication space even beyond autoimmune diseases is addressed in this review.

Herein, we focus on reviewing how first-generation TCE formats are being modified to become next-generation TCEs with improved properties. A key learning from the twelve approved, first-generation TCEs is that a wide variety of TCE formats can succeed and lead to regulatory approval (1). The majority of commercial TCEs use asymmetric IgG-like molecules where the two arms of the antibody are different. Asymmetry requires technologies for heterodimerization of the IgG’s Fc domain such as knob-in-hole or IgG4-based duobodies. The two first approved TCEs, blinatumomab and tebentafusp, leveraged small single-chain Fv or soluble TCR fragments, respectively, and did not include domains for serum half-life extension. The resulting short serum half-life necessitated continuous dosing of blinatumomab via an intravenous port, both inconvenient and prone to infections (1). Most currently approved TCEs preferentially utilize IgG-like formats (17), enabling serum half-life extension via the Fc domain, which binds FcRn, a neonatal receptor that prolongs immunoglobulin serum half-life (**Figure 1A**). The Fc domain is typically silenced by point mutations to disable binding to Fc gamma receptors --which could otherwise activate innate immune mechanisms to deplete the engaged T cells. Instead of an Fc portion, emerging TCEs may alternatively use a small serum albumin-binding VHH domain for half-life extension (18).

**Figure 2:**
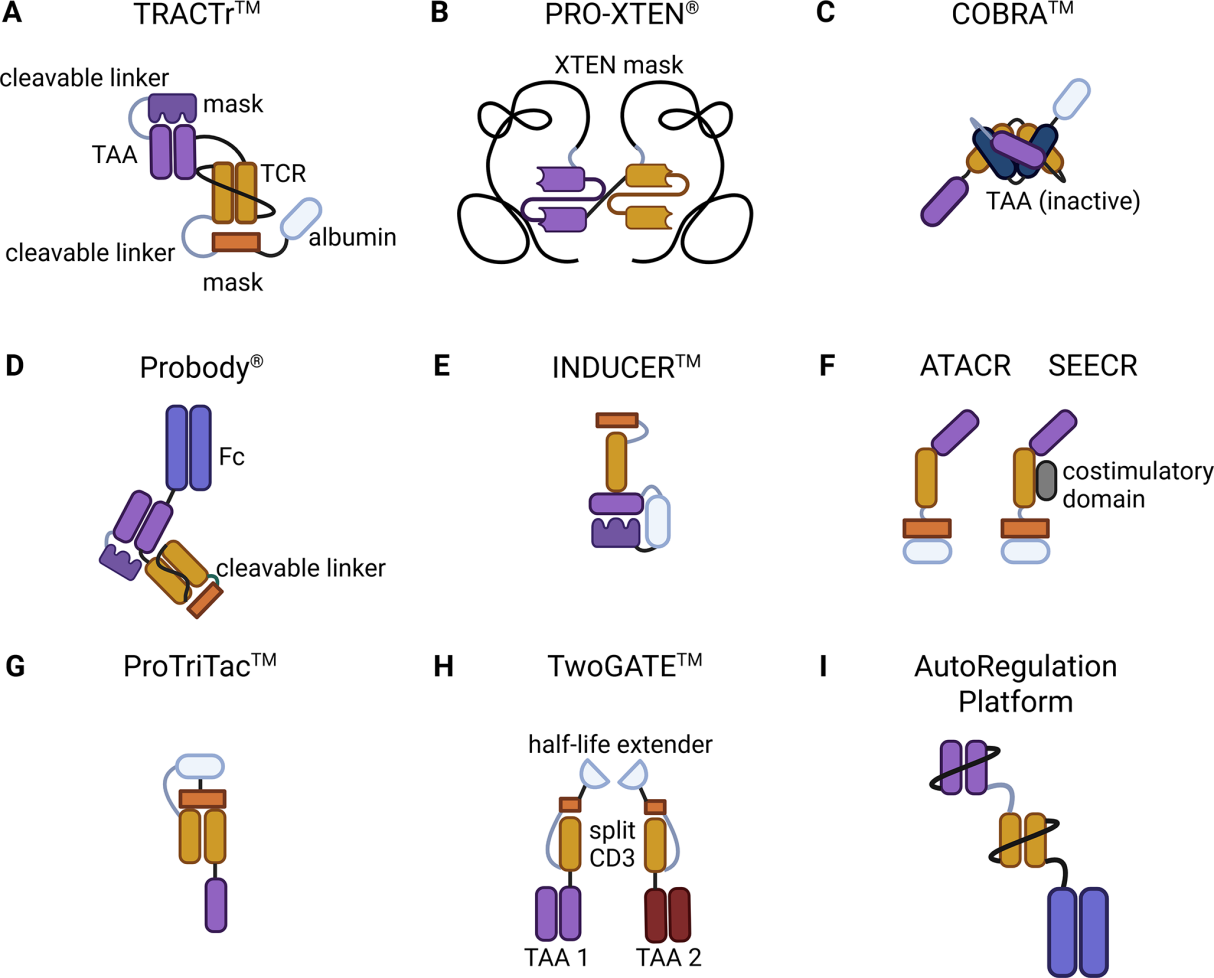
Examples of TCEs Conditionally Activated by TME Proteases. **(A)** Tumor Activated T Cell Engagers (TRACTr™) consist of a binder to a tumor associated antigen (“TAA”) and a binder to the T cell receptor (“TCR”), and an albumin binder for half-life extension. The TCR binder is masked and, in some instances, the TAA binder is also masked, such as with JANX014. The protease cleavable linker connects one of the masks to the albumin binder (19). **(B)** Protease-releasable masking technology (PRO-XTEN^®^) uses a PEG-like peptide mask fused to the TAA and T cell single chain variable fragments (scFvs) via protease cleavable linkers to enable half-life extension and prevent target engagement until the masks are removed (7). **(C)** Conditional Bispecific Redirected Activation (COBRA™) TCEs are comprised of a single chain molecule which links two TAA binders, separated by anti-CD3 variable heavy and light chains, to inactive variable heavy and light chains and a human serum albumin (HSA) binder via a protease cleavable linker. The constrained linkers between the variable heavy and light chains prevent the formation of active CD3 scFvs until exposure to proteases (20). **(D)** CX-908, a Probody^®^ therapeutic, utilizes protease cleavable masks for the TAA and T cell receptor binder and a silenced Fc for half-life extension (21). **(E)** INDUCER™ molecules utilize two identical protease cleavable linkers, one of which is connected to the anti-CD3 antibody mask and the other fused to a subunit consisting of an anti-HSA VHH and TAA targeting mask (22). **(F)** Advanced Tumor-Activation Cell Engager (ATACR) utilizes a protease cleavable linker to attach a subunit containing a masking domain and half-life extension domain to a CD3 binder. Selective Effector-Enhanced Cell Engager (SEECR) has the same format as ATACR with the addition of a costimulatory domain connected to the CD3 binder (23). **(G)** For ProTriTac™ TCEs, the CD3 mask is attached to a HSA binder, which is connected to the CD3 binder through a protease cleavable linker (24). **(H)** Two component Guided Antibody Engager (TwoGATE™) consists of two independent components targeting different TAAs (TAA1 and TAA2), each of which has a half-life extender and a CD3 mask subunit connected to a split paratope CD3 binder via a protease cleavable linker (25); **(I)** The AutoRegulation Platform connects the TAA binder to a Fc half-life extended T cell binder via a peptide linker which is susceptible to granzyme B cleavage when the protease is present at high concentrations (26). Created in BioRender. Henry, C (2026). https://BioRender.com/wrsahel.

**Figure 3:**
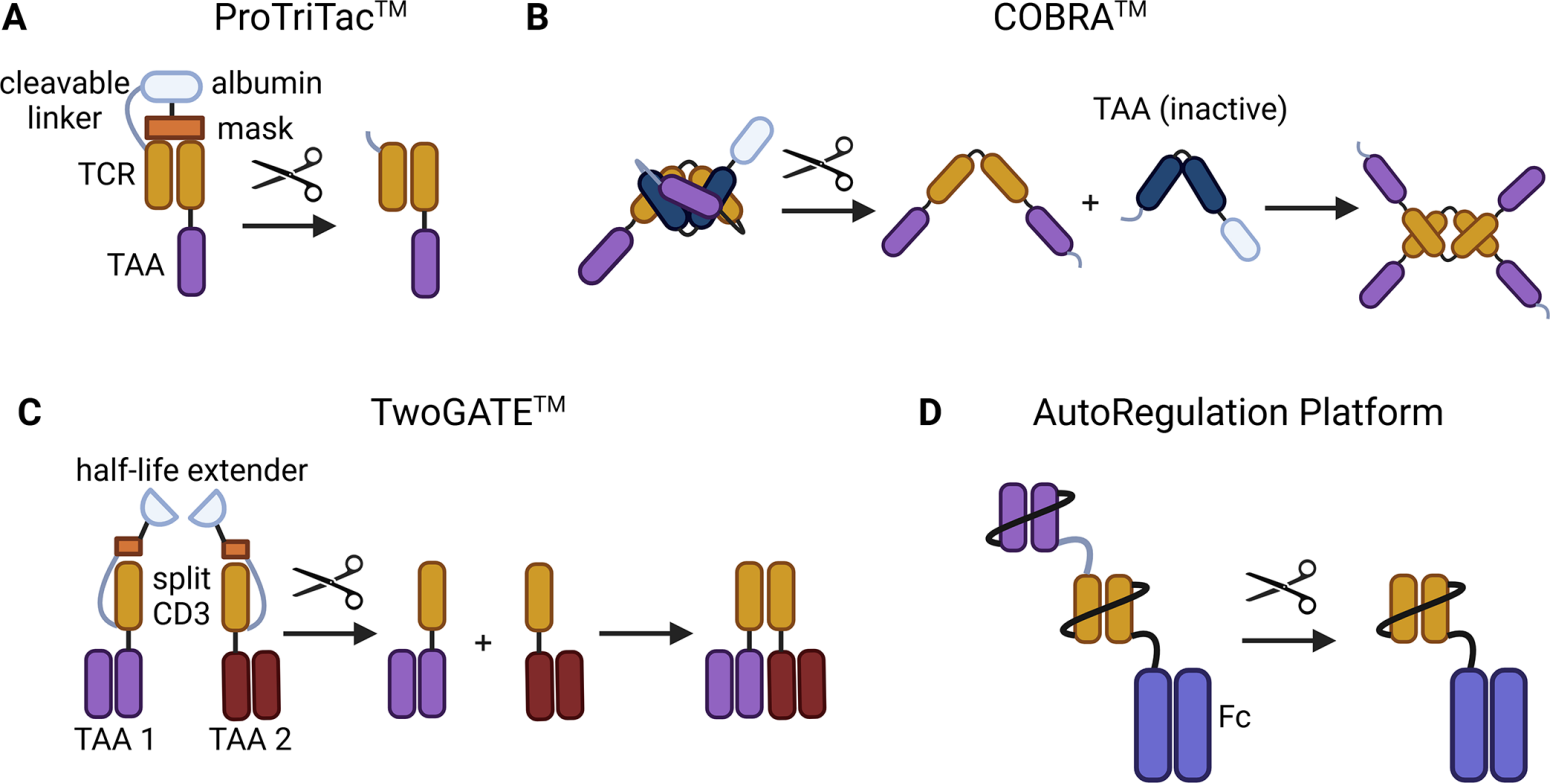
Mechanism of action for TCEs conditionally activated by TME proteases. **(A)** Schematic showing active TCE formation of a ProTriTac™ following protease cleavage which removes the masking and half-life extension domains. This mechanism of action is similar for TRACTr™, PRO-XTEN^®^, Probody^®^, INDUCER™, ATACR, and SEECR formats, although Probody^®^ TCEs retain their Fc domain following protease cleavage. **(B)** Upon protease cleavage, COBRA™ molecules are split into monomers that bind TAA, as well as an anti-HSA subunit bound to inactive variable heavy and light domains. A functional CD3 binder is only formed when the monomers dimerize, resulting in an active TCE (20). **(C)** TwoGATE™ constructs form split paratope CD3 binders bound to TAA following protease cleavage. To enable T cell mediated killing, the paratopes must associate to form a functional anti-CD3 binder and the two TAAs must bind their respective antigens (25). **(D)** For the AutoRegulation Platform, the TCE is active until a threshold of granzyme B protease is reached, resulting in protease cleavage between the TAA and CD3 binders, which renders it inactive. Scissors indicate protease cleavage. Created in BioRender. Henry, C (2026). https://BioRender.com/wrsahel.

**Figure 4:**
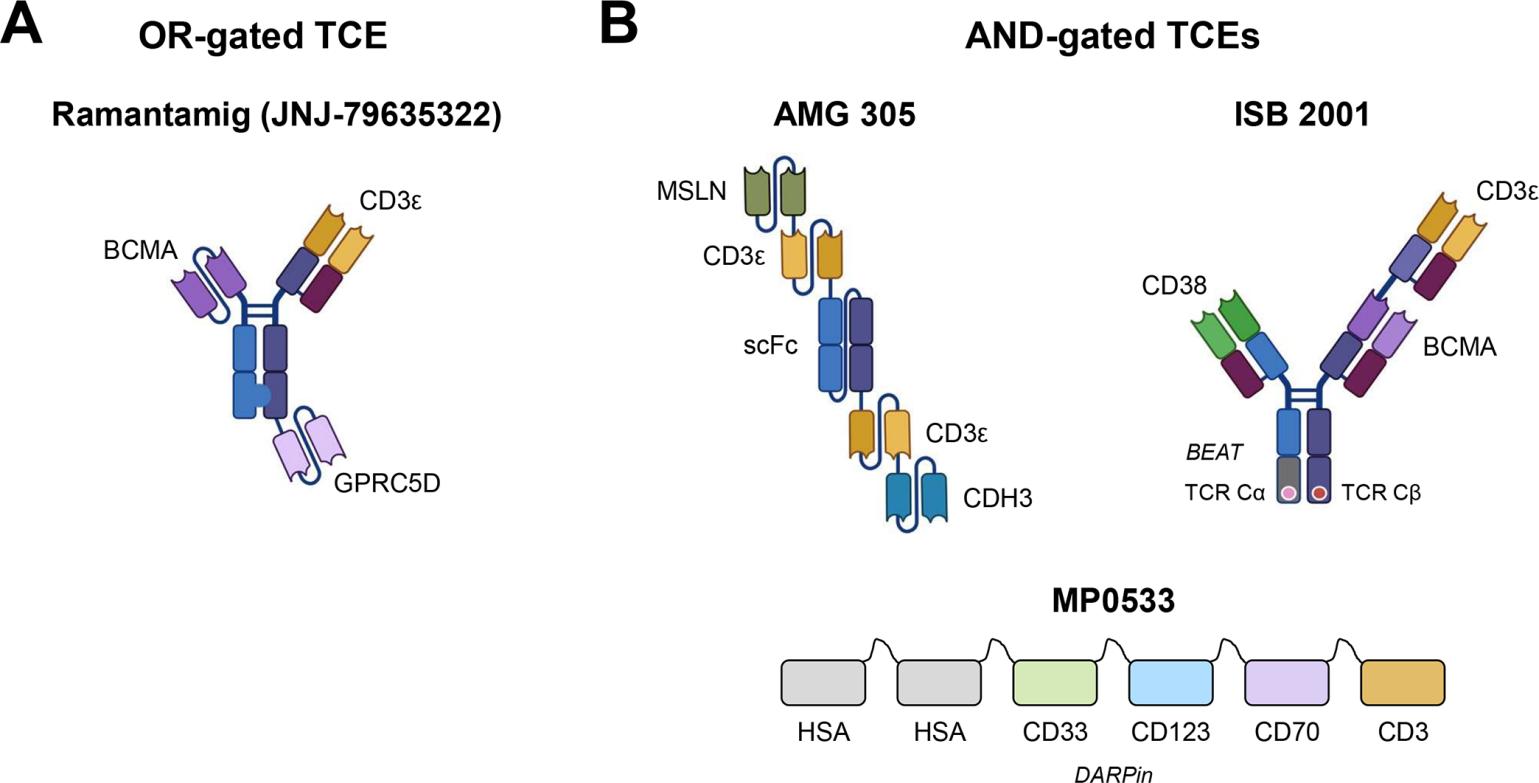
Examples of logic-gated TCEs. **(A)** A clinical example of an OR-gated TCE is Ramantamig (JNJ-79635322) (27). It is based on an asymmetric IgG Fc stabilized by knock-in-hole mutations and employs a Fab-domain for CD3ϵ-binding on T cells and two different single-chain Fv (scFv) moieties that bind the target antigens BCMA and GPRC5D on plasma cells and MM cells. The BCMA scFv replaces the second Fab-domain while the GPRC5D scFv is linked to the C-terminal base of the Fc-region (28). Binding of either target antigen triggers activation of the bound T cell. **(B)** Three clinical examples illustrate that diverse formats can be leveraged for AND-gated TCEs. Both target antigens must be bound to trigger activation of the bound T cell, whereas binding of only one antigen has little or no effect. AMG 305 is a BiTE based on a single-chain Fc (scFc) region to which two tandem-scFvs are attached at each end. The tandem scFvs each contain one scFc-proximal binder for CD3ϵ and a distal binder for the solid tumor target antigens MSLN or CDH3 (27, 29). ISB 2001 is a Bispecific Engagement by Antibodies based on the TCR (BEAT) format TCE with Fab-based binders for CD3ϵ and for the plasma cell/MM cell target-antigens BCMA and CD38. The CD3ϵ and BCMA binders are linked in tandem on one arm of the BEAT. The BEAT-format is based on an IgG where one heavy chain contains engineered human IgG1 CH2 and IgG3 CH3 domains, while the other heavy chain contains engineered human IgG1 CH2 and CH3 domains. Pink and red dots denote BEAT interface proprietary mutations based on the TCR constant domain α or β, respectively (27, 30). MP0533 is a multispecific, single-polypeptide, designed ankyrin repeat protein (DARPin) that can simultaneously bind to CD3 on T cells and to three antigens on AML cells, aiming to enable avidity-driven killing of AML cells coexpressing at least two of the antigens. Additional DARPin domains include two N-terminal HSA-binders for half-life extension (31). Created partly in BioRender. Sauer, K (2026). https://BioRender.com/b0yl9kw.

**Figure 5:**
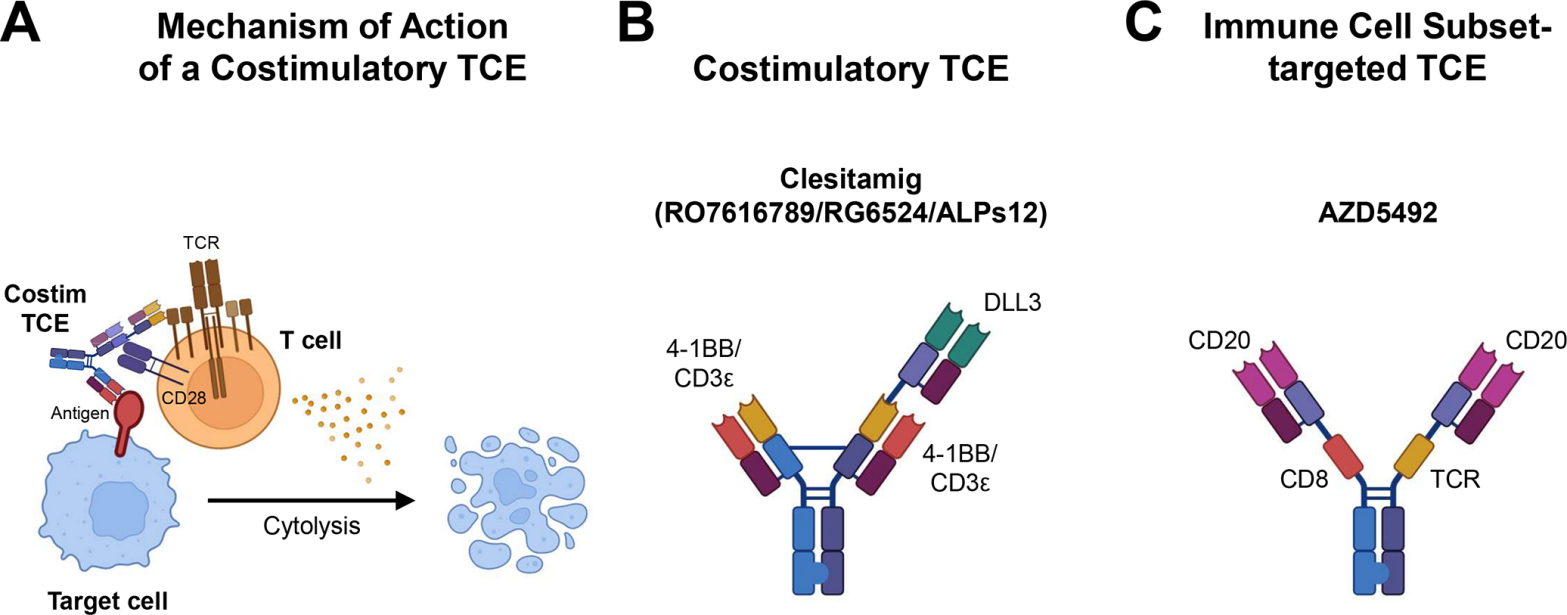
Mechanism of action of and examples for costimulatory and T cell subset-targeted TCEs. **(A)** Costimulatory TCEs bind to a target antigen on a target cell and to both a TCR-subunit (typically CD3ϵ) to provide signal 1 and a costimulatory receptor (here CD28) to provide signal 2 on a T cell. This activates the T cell to secrete cytolytic granules and thereby kill the target cell. Costimulation synergizes with TCR-engagement and may prevent activation-induced T cell death or induction of T cell anergy or exhaustion (27, 32). **(B, C)** Published formats of a costimulatory and a CD8 T cell subset-targeted TCE (27). **(B)** The costimulatory TCE clesitamig represents an asymmetric, knob-in-hole stabilized 2 + 1 IgG where two dual-specificity Fab-fragments competitively bind CD3ϵ and costimulatory 4-1BB on T cells and a third Fab binds the antigen DLL3 on target cells. To prevent trans-binding to two different T cells and resulting fratricide, a disulfide bond was introduced between the two CD3ϵ/4-1BB binders (33). **(C)** The CD8 T cell-targeted TCE AZD5492 is an asymmetric, knob-in-hole stabilized IgG1 whose antigen-binding arms contain tandem-arrangements of two distal Fab-fragments binding the B cell antigen CD20, each with a Fc-proximal VHH binder for the TCR or CD8, respectively (27, 34). Created partly in BioRender. Sauer, K (2026). https://BioRender.com/b0yl9kw.

Major approaches for and advances in developing next-generation TCEs include optimization of binding affinities, particularly for binding the TCR; incorporation of conditional activation of the TCE in the tumor microenvironment (TME), logic gating for multi-target specific TCEs, or addition of T cell costimulatory domains providing signal 2; engagement of specific subpopulations of T cells to minimize toxicities; and broadening of the indication space for TCEs into additional solid tumor indications and autoimmune diseases, and beyond. Each of these approaches will be described and discussed in this review. **Figures 1**–**5** show examples of first generation TCEs and next-generation TCE formats.

## Affinity optimization of TCEs

An obvious approach to improving TCE efficacy and safety is optimizing the affinities of binding elements to CD3ϵ and the target antigen. This has been addressed in TCE drug design from the inception. The first approved TCE, blinatumomab, used a very low affinity for binding CD3ϵ of K_D_ = 250 nM and a much higher affinity for CD19, in the range of K_D_ =1 nM. However, subsequently approved TCEs all had higher affinities for CD3 with ratios of affinities for CD3 to target binding ranging over more than 3 logs. From the first twelve approved TCEs, there was no apparent trend for selecting affinities (1).

What appears most desirable is to select TCE candidates featuring a ‘cytokine window’, i.e., a robust difference between TCDD activity and cytokine release. If redirected lysis occurs at TCE concentrations lower than those which induce the release of cytokines, such as TNF-α or interferon-γ, then the TCE may lead to less high-grade CRS at efficacious dose levels in the clinic. This concept prompted the incorporation of very low-affinity CD3 binding elements into TCE design. Preclinical data supported that cytokine release was much diminished, however so was the potency of TDCC (35). When one such CD19/CD3 bispecific TCE (TNB-486) was taken into clinical trials, it required much higher dosing to achieve efficacy, which then yielded grade 3 CRS and ICANS and the requirement for a multiple step-up dosing regimen (36, 37). A PSMA-specific TCE with a very low affinity CD3 binder (TNB-588) was developed but discontinued after a phase 1 clinical study in 2023 for undisclosed reasons (38). Indeed, it is likely that some cytokine release may be required to enable TCEs to cause bystander killing of antigen-negative cells although the clinical relevance of this mechanism for anti-tumor activity remains unclear (39–41). Taken together, these examples highlight apparent tradeoffs from using very low affinity CD3 binders yielding less optimal clinical outcomes that may be a result of insufficient T cell activation or infiltration or compromised antitumor activity, thus underscoring the delicate balance between safety and efficacy that requires optimal tuning of affinities.

Another critical aspect of affinity optimization relates to target binding. Multivalent binding of tumor-associated antigens (TAAs) has the potential to modulate overall T cell functional potency by leveraging avidity effects. TCEs constructed with two binders targeting the same or two different epitopes on a given TAA can be a means to increase apparent binding affinity via avid target binding. The same is true for binding two or more distinct TAAs by a multi-targeted TCE (e.g. OR-gated TCEs, see below). In all cases, careful design of the TCE format and selection of optimal binding affinities for TAA-binding arms as well as the CD3-binding arm is necessary.

## Conditionally activated TCEs

While TCEs are inherently conditional, as they require the simultaneous engagement of target and CD3 for activity, further conditional activation specific to the tumor microenvironment (TME) is an emerging strategy for overcoming the dearth of tumor-specific antigens in solid tumors. TCEs can kill cells expressing as few as 10 copies of target (1, 5, 42). While this is a major advantage of the modality for highly tumor specific antigens, there is a scarcity of tumor restricted antigens, particularly for solid tumors, with many targets being “dirty” in that they have some level of expression on critical normal tissues resulting in on-target/off-tumor toxicities. Unlike solid tumor therapies, therapeutics for hematologic malignancies can leverage lineage-specific targets to deplete malignant cells along with the entire normal cell lineage (e.g., B cells) because transient depletion of many hematopoietic cell types is manageable and they can eventually be regenerated from bone marrow progenitors. The more restrictive tissue expression requirements for solid tumor targets likely account for why only 2 of the 9 indications in which TCEs are approved in the US are for solid tumor indications. Notably, gp100 and DLL3, the targets of Tebentafusp approved in uveal melanoma and Tarlatamab approved in extensive-stage small cell lung cancer, respectively, are both highly tumor-selective targets (42, 43). Clinical trials with TCEs that have targeted “dirty” antigens, such as EpCAM (44) and CEACAM5 (45, 46) have been discontinued, perhaps at least in part due to dose limiting toxicities associated with expression of these targets.

### Protease-cleavable TCEs

To broaden the target space, technologies have been developed to restrict activation of TCEs to the TME. One characteristic that distinguishes normal and tumor tissue is the high abundance of various proteases within tumor tissue, which facilitate tumor progression, metastasis, extracellular remodeling, and inflammation (47, 48). A preferred method for conditional activation of TCEs is the fusion of a proteolytically removable mask that shields the TAA and/or the CD3 binder. Most platforms typically fuse the mask via a linker sequence that contains multiple protease cleavage sites, obviating the need to identify specific proteases in the TME of tumor indications. Upon proteolytic activation of the TCE in the TME, not only is the binding site exposed but often a half-life extending domain is released ensuring that the TCE is rapidly cleared from circulation following activation (**Figures 2**, **3A**) (7, 19, 21, 23, 49–53).

Some protease-masking technologies are engineered with more elaborate modes of conditional activation. For example, following protease cleavage, Revitope’s Two component Guided Antibody Tumor Engager (TwoGATE™) (25) and Takeda’s Conditional Bispecific Redirected Activation (COBRA™) platforms (54) require a dimerization of subunits to generate a fully active TCE (**Table 1**; **Figures 3B, C**). Another conditional activation strategy incorporates protease cleavage linkers into the construct as a safety switch (26, 70), whereby exposure to high levels of granzyme B, which is released by activated T cells, will limit their activity via proteolysis.

**Table 1:** Conditional TCEs activated by TME proteases.

Company	Partner	Assets	Platform	Indication	Phase	Trial identifier	References
Janux	N/A	JANX007 (PSMAx**CD3**)	TRACTr™	mCRPC	Ph 1	NCT05519449	(49)
	N/A	JANX014 (**PSMA**x**CD3**)		mCPRC	Ph 1	NCT07545811	(19)
	N/A	JANX008 (**EGFR**x**CD3**)		EGFR+ Solid tumors	Ph 1 (Discontinued)	NCT05783622	(50)
	Merck	2 Undisclosed programs		Undisclosed	Ph 1/Undisclosed	Unknown	(19)
	Bristol Myers Squibb	Undisclosed program		Undisclosed	Preclinical	N/A	(19)
Vir	Sanofi	VIR-5818 (**HER2xCD3**)	PRO-XTEN^®^	HER2+ Solid tumors	Ph 1	NCT05356741	(7)
	Sanofi	VIR-5525 (**EGFRxCD3**)		EGFR+ Solid tumors	Ph 1	NCT06960395	(7, 55)
	Astellas, Sanofi	VIR-5500 (**PSMAxCD3**)		mCRPC	Ph 1	NCT05997615	(56)
	N/A	Undisclosed TCEs		Solid tumors	Preclinical	N/A	(57)
Takeda	N/A	TAK-186 (EGFRx**CD3**)	COBRA™	EGFR+ Solid tumors	Ph 2 (Discontinued)	NCT04844073	(20, 58)
	N/A	TAK-280 (B7H3x**CD3**)		B7H3+ Solid tumors	Ph 1/2 (Discontinued)	NCT05220098	(59, 60)
Cytomx	N/A	CX-908 (**CDH3**x**CD3**)	Probody^®^	Solid tumors	Preclinical	N/A	(21, 51)
	Regeneron	Undisclosed		Solid tumors	Preclinical	N/A	(61)
	Bristol Myers Squibb	Undisclosed		Solid tumors	Preclinical	N/A	(61)
	Amgen	CX-904 (**EGFR**x**CD3**)		Solid tumors	Ph 1 (Discontinued)	NCT05387265	(62, 63)
	Astellas	Multiple undisclosed		Solid tumors	Preclinical (Discontinued)	N/A	(64)
Werewolf	N/A	WTX-1011 (**STEAP1**x**CD3**)	INDUCER™	STEAP1+ Cancers	Preclinical	N/A	(22, 52)
	N/A	WTX-2022 (**CDH6**x**CD3**)		CDH6+ Cancers	Preclinical	N/A	(22)
	N/A	Multiple undisclosed		Cancer	Preclinical	N/A	
Xilio	N/A	STEAP1xPSMAx**CD3**	SEECR	Prostate Cancer	Preclinical	N/A	(23, 65, 66)
		XTX601 (CLDN18.2x**CD3)**	ATACR	Solid tumors	Preclinical	N/A	
	AbbVie	Undisclosed	Undisclosed	Undisclosed	Preclinical	N/A	(65)
Merck	N/A	HPN601 (EpCAMx**CD3**)	ProTriTac™	Solid tumor	Preclinical (status unknown)	N/A	(53)
	N/A	ITGB6x**CD3**		ITGB6+ Solid tumors	Preclinical (status unknown)	N/A	(67)
	N/A	TROP2x**CD3**		TROP2+ Solid tumors	Preclinical (status unknown)	N/A	(68)
Revitope	N/A	REV-403 (EGFRxPDL1x**CD3)**	TwoGATE™	Solid tumors	Preclinical	N/A	(25, 69)
	Junshi	Multiple undisclosed		Solid tumors	Preclinical	N/A	
	Genmab	Multiple undisclosed		Solid tumors	Preclinical	N/A	
NovalGen	N/A	NVG-111 (ROR1xCD3)	AutoRegulation (AR) platform	Heme malignancies/Solid tumors	Ph 1 (Unknown status)	NCT04763083	(55, 70)
	Cancer Research UK	NVG-222 (ROR1xCD3xFc)		Heme malignancies/Solid tumors	Ph 1/Preclinical	ISRCTN14974342	(26, 71)
	Cancer Research UK	NVG-666 (CD19xCD3xFc)		Autoimmune disease	Preclinical	N/A	(71)
	N/A	2 Multi-specifics programs		Undisclosed	Preclinical	N/A	

Bold font indicates a moiety of the asset that is masked directly or indirectly. PSMA, prostate-specific membrane antigen; CD3, cluster of differentiation 3; TRACTr, tumor activated T cell engager; mCRPC, metastatic castration resistant prostate cancer; EGFR, epidermal growth factor receptor; HER2, human epidermal growth factor receptor 2; PRO-XTEN, protease-releasable masking technology; COBRA, conditional bispecific redirected activation; B7H3, B7 homolog 3 protein; CDH3, cadherin-3; STEAP1, six-transmembrane epithelial antigen of the prostate 1; CDH6, cadherin-6; SEECR, selective effector-enhanced cell engager; ATACR, advanced tumor-activated cell engager; CLDN18.2, claudin 18.2; EpCAM, epithelial cell adhesion molecule; ProTriTac, pro-tri-specific T cell activating constructs; ITGB6, integrin-β6; TROP2, trophoblast cell-surface antigen 2; PDL1, programmed death-ligand 1; ROR1, receptor tyrosine kinase-like orphan receptor 1; Fc, fragment crystallizable; CD19, cluster of differentiation 19.

To date, Vir Bio has provided the most compelling clinical data with their PRO-XTEN^®^ platform. It utilizes a protease-cleavable, polyethylene glycol-like polypeptide, which can provide both half-life extension as well as comprehensive shielding of both the TAA and CD3 binding sites of the TCE. In metastatic castration resistant prostate cancer (mCRPC) patients, treatment with VIR-5500 targeting prostate-specific membrane antigen (PSMA) led to a ≥90% reduction in prostate-specific antigen (PSA), a soluble biomarker for prostate cancer, in 53% of patients and a 45% overall response rate (ORR) (56, 72). Additionally, VIR-5818, a TCE targeting human epidermal growth factor receptor 2 (HER2), has shown early signs of efficacy (33% ORR) and acceptable safety in six heavily pretreated HER2+ colorectal cancer patients (73). JANX007, another TCE with a masked CD3ϵ binding domain targeting PSMA, has also shown signs of clinical activity in mCRPC (30% ORR in 27 patients) (19, 74). It is now followed with a double-masked version, called JANX014. However, more clinical data is needed to conclusively assess the efficacy of these protease-cleavable TCEs. Other conditional TCEs have shown limited activity in the clinic leading to trial discontinuations, including CytomX’s CX-904 (62) and Takeda’s COBRA TCEs (75–78). Thus far, toxicity signals have not been cited for any clinical discontinuation. One reason for mixed clinical results could be the heterogeneity of protease profiles across indications and within tumors and patient populations which may reduce the generalizability and efficacy of protease-activatable TCEs (75–77). Another reason for the clinical discrepancies could be differences in molecular architectures of the various platforms. For example, CX-904 maintains its Fc domain following unmasking, potentially allowing for on-target, off-tumor engagement outside the TME, whereas assets with positive clinical signals, such as those developed by Vir and Janux, lose their half-life extension domain upon protease cleavage allowing for rapid clearance in the periphery. Additionally, Takeda’s COBRA TCEs may have suffered clinically due to the added complexity of requiring dimers to form following protease cleavage to generate an active TCE.

### pH-activated TCEs

Another emerging strategy for conditional activation of TCEs is the use of pH-sensitive binders for the TAA or CD3. In contrast to the typical pH of ~7.4 in normal tissues (79), the pH in the TME is usually more acidic at pH values between 5.3 and 7.0 due to the high dependence of cancer cells on glycolysis and the resulting release of lactic acid (80–82). Binders can be engineered to only engage the TAA at low pH by introducing charged amino acid substitutions into their complementarity-determining (CDR) regions that do not interfere with antigen specificity. Under normal physiological conditions, these amino acids noncovalently interact with bicarbonate or hydrogen sulfide ions to block target binding and act as a chemical switch depending on environmental pH (83, 84). Currently, BA3182, a dual activation EpCAM/CD3 TCE developed by BioAtla, is the only pH activated TCE in clinical development (**Table 2**) (92). Preliminary results from a phase 1 trial suggest evidence of anti-tumor activity with acceptable tolerability (85).

**Table 2:** TCEs activated by a low pH.

Company	Asset	Platform	Indication	Status	Trial Identifier	References
BioAtla	BA3182 (**EpCAM**x**CD3**)	CAB™	Adenocarcinomas	Ph 1	NCT05808634	(85, 86)
	BA3142 (**B7H3**x**CD3**)		Solid tumors	Preclinical		(86, 87)
	BA3311 (EGFRx**CD3**)		Solid tumors	Preclinical		(86)
	BA3241 (**TROP2**x**CD3**)		Solid tumors	Preclinical		(86)
Context	CT-202 (BA3362) (**Nectin-4xCD3**)		Solid tumors	Preclinical		(86, 88)
Amberstone	ABS-106 (STEAP1x**CD3**)	T-MATE^®^	Solid tumors	Preclinical		(89–91)
	ABS-10X (TAAx**CD3**)		Unknown	Preclinical		(89, 90)

Bold font indicates a portion of the asset that is pH dependent. EpCAM, epithelial cell adhesion molecule; CD3, cluster of differentiation 3; CAB, conditionally active biologic; B7H3, B7 homolog 3 protein; EGFR, epidermal growth factor receptor; TROP2, trophoblast cell surface antigen-2; STEAP1, six-transmembrane epithelial antigen of the prostate 1; T-MATE, tumor micro-environment activated therapeutics; TAA, tumor associated antigen.

While pH-activated TCEs have seen less clinical validation to-date compared to proteolytically activated TCEs, the more consistently observed phenomenon of acidic pH compared to protease heterogeneity in the TME may play in their favor (80–82, 93). Other potential advantages of pH-activated TCEs include reduced molecular complexity and reversible activation, which could enable improved developability profiles and provide more controlled activation profiles, respectively. However, there are potential challenges in preclinical development, such as optimizing multi-day T cell assays in low pH conditions (94–96), as well as further development challenges when targeting antigens present on normal tissues with lower pH at homeostasis (e.g., lung, stomach) which may require narrow, precisely engineered pH activation profiles (97). It is also unclear whether micrometastases exhibit acidic pH profiles similar to those of larger tumors.

Elevated levels of extracellular ATP and β−glucuronidase activity are additional hallmarks of the TME that may be exploited for conditional activation of TCEs (98–101), but no such conditionally activated TCEs are currently in clinical development (102–104). The application of conditional activation currently pursued in oncology for the development of TCEs in autoimmune indications will also require future investigation. Although there are some autoimmune diseases, such as RA, which have locally increased proteolytic enzymes (105, 106) and low pH (107), most other indications are systemic in nature. Pathogenic immune cells may need to be depleted in lymph nodes and other tissues, where the environment may differ from that in diseased tissues, and potentially even in systemic circulation.

## Logic-gated TCE designs

A particular challenge of any targeted cancer therapy is heterogenous expression of the target antigen. Cancer cells expressing very low levels of target antigen may escape targeted therapy and become the seeds for emerging tumor clones selected for no or very low target expression. Likewise, target-negative cancer cells can be selected following exposure to mAbs, ADCs or TCEs. TCEs, like CAR-T cells, can effectively induce cytolysis of target-negative bystander cells through mechanisms involving cytokine release and *de novo* expression of Fas receptor and Fas ligand (39–41). However, while capable of eliminating nearby target-negative cells, bystander killing is unlikely to eradicate larger areas of target-negative cancer cells inside tumors. One approach to address this challenge may be the combination of TCEs with therapeutics employing an orthogonal mode of action.

For TCEs and other antibody-based therapies, a unique approach is to engineer a single therapeutic that can recognize more than one target antigen via logic gating (27). There are two principally distinct mechanisms for dual- or multi-targeting of TCEs. One is that each target antigen is independently recognized by the TCE, and TDCC can occur by binding one or the other TAA or both, on a cancer cell. This is referred to as ‘OR-gating’ (27). No OR-gating TCEs have thus far achieved regulatory approval, however, multiple candidates are in early clinical development (**Table 3**). The promise of OR-gating TCEs is to achieve higher response rates (RRs) by mitigating the challenges of heterogenous target expression and by leveraging the higher target copy numbers for binding that are enabled by targeting more than one antigen. OR-gating also reduces the risk of tumor escape via downregulation of oneTAA. However, one challenge of OR-gating is the requirement for both targets to be highly tumor specific, since toxicities will be cumulative.

**Table 3:** Logic-gated TCEs in clinical development.

Gating strategy	Targets	Company	Asset	Indication(s)	Phase	Trial ID	References
AND	MSLNxCDH3xCD3	Amgen	AMG 305	ST	1 (completed)	NCT05800964	(29, 55)
	HSAxCD33xCD123xCD70xCD3	Molecular Partners	MP0533 (DARPin)	AML	1/2a	NCT05673057	(31, 55, 108)
	BCMAxCD38xCD3	Ichnos Sciences SA/AbbVie	ISB 2001	MM	1	NCT05862012	(30, 55, 109, 110)
OR	BCMAxGPRC5DxCD3	Janssen R&D	Ramantamig (JNJ-79635322)	MM, AL Amyloidosis	1	NCT05652335, NCT06768489, NCT07266441, NCT07258511, NCT07518186	(28, 55, 111)
	BCMAxGPRC5DxCD3	Qilu Pharmaceutical Co.	QLS4131	MM, SLE	1	NCT06500507, NCT07001839	(55, 112)
	BCMAxGPRC5DxCD3	Jiangsu Simcere Pharmaceutical Co./AbbVie	SIM0500	MM	1	NCT06375044	(55, 113, 114)
	CD19xCD20xCD3	Chimagen Biosciences/GSK	CMG1A46	NHL	Ph 1/2 terminated post acquisition by GSK, planned development in SLE/LN	NCT05348889	(55, 115, 116)
	CD79bxCD20xCD3	Janssen R&D	JNJ-80948543	NHL, CLL	1	NCT05424822, NCT06660563, NCT06139406, NCT06788509	(55, 117)
	CD79bxCD20xCD3	Janssen R&D	JNJ-95566692	NHL	1	NCT07308132	(55)
	CD79bxCD20xCD3	Qilu Pharmaceutical Co.	QLS2313	Hematological Malignancies	1	NCT07294300	(55, 118)
	PD-L1xHLA-GxCD3	Shine-On Biomedical Co.	SOA101	ST	1/2a	NCT07055594	(55, 119)
	MTS-128 (CD19xBCMAxCD3)	METiS TechBio	mRNA TCE	Cancer, AID	Preclinical	N/A	(120)

AID, autoimmune disease(s); AL amyloid light chain amyloidosis; AML, acute myelogenous leukemia; BCMA, B cell maturation antigen; CD123, cluster of differentiation 123/interleukin-3 receptor; CD19, cluster of differentiation 19; CD20, cluster of differentiation 20; CD3, cluster of differentiation 3; CD33, cluster of differentiation 33/Siglec-3; CD38, cluster of differentiation 38; CD70, cluster of differentiation 70; CD79b, cluster of differentiation 79b/immunoglobulin-associated beta; CDH3, cadherin-3; CLL, chronic lymphocytic leukemia; DARPin, designed ankyrin repeat protein; GPRC5D, G-protein coupled receptor family C group 5 member D; HLA-G, human leukocyte antigen G; HSA, human serum albumin; MM, multiple myeloma; MSLN, mesothelin; NHL, Non-Hodgkin lymphoma; PD-L1, programmed death-ligand 1; SLE, systemic lupus erythematosus; ST, solid tumor.

The initial emerging data suggests that OR–gating can indeed lead to higher RRs in the treatment of MM. The TCE ramantamig (JNJ-79635322) which co-targets BCMA and GPRC5D (**Figure 4A**) achieved an ORR of 100% in relapsed/refractory MM patients not previously treated with cell-depleting therapies (111). This exceeds the ORRs observed with single-targeting TCEs or after subsequent treatment with two TCEs, suggesting that dual-targeting TCEs may create unique binding properties favoring T cell engagement and TDCC.

The alternative principle for dual targeting is referred to as ‘AND-gating’ (27). Here, TCEs only sub-optimally bind to either target, but require simultaneous binding to both targets for optimal TDCC. This introduces conditionality in that the TCE action is dependent on the co-expression of tumor target pairs, importantly with minimal or no co-expression on the same normal tissue. AND-gating affords some leniency with regard to selection of tumor targets and may be a means to widen the therapeutic window of the TCE, enabling incorporation of a broader range of target antigens. A disadvantage of AND-gating is the need for target pairs that are frequently co-expressed on cancer cells, which can reduce the addressable patient population and exacerbate the impact of heterogenous target expression. AND-gating TCEs are typically more challenging to develop as compared to OR-gated TCEs since the affinities need to be finely tuned and optimally selected to enable appropriate AND-gated binding properties. The requirement to select ultra-low affinity binders for each target to achieve cooperative dual target engagement can require extensive screening to identify the optimal pair of binders. An alternative strategy to achieve AND-gating is employment of a split domain approach (121), such as Revitope’s “*Two*GATE” technology, which relies on two distinct components to each bind to its respective target and then assemble to generate an active TCE *in situ* (**Figure 3C**). While elegant, this introduces additional complexity including the requirement for developing two distinct drug candidates.

One example of a cooperatively binding AND-gated mAb is amivantamab, co-targeting EGFR and cMet. It is approved for the treatment of EGFR mutant non-small cell lung cancer (122). EGFR is widely expressed on normal tissues including liver, lung and skin and known to cause side effects from off tumor/on target binding. However, AND-gating introduces the dependence on cMet binding, whereby toxicities from targeting EGFR-expressing normal tissues are largely dialed out. Several AND-gating TCEs are in clinical development but to-date none have progressed to registrational studies (**Figure 4B**; **Table 3**). The lack of CRS above grade 2, ICANS or DLTs, and the reported ORR of 89.5% for the AND-gated BCMAxCD38xCD3 TCE ISB 2001 are encouraging (30, 109). A more definitive comparison to monospecific TCEs and to multi-targeted TCEs that are not AND-gated awaits larger clinical studies. Multi-targeting is also a trend in the development of other next-generation antibody-based therapies, including mAbs, ADCs and CAR-T cells (27, 123).

In summary, OR-gated TCEs provide a means to address clonal heterogeneity and avoid target antigen escape, whereas AND-gated systems provide a solution for addressing targets that would otherwise suffer from on-target, off-tumor toxicities.

## TCEs with costimulatory domains

Another approach to multitargeting is the addition of domains to TCEs that agonize costimulatory T cell receptors, such as CD28, 4-1BB/CD137 or CD2 (**Figure 5A**) (124). Canonical TCEs typically provide only signal 1, i.e., TCR activation, for engagement of T cells (**Figure 1B**). This selectively activates and expands effector memory T cells, a phenotype largely independent of costimulation. Occasionally, target cells provide signal 2 by naturally expressing agonistic ligands for costimulatory T cell receptors. For example, antigen-presenting cells or certain tumor cells express 4-1BBL, the ligand for 4-1BB (32). Preclinical data have shown that providing signal 2 can augment TDCC and counter T cell exhaustion as seen with the limited number of T cells typically used in cell co-culture assays (32). While signal 2 may not impact T cells that have lost costimulatory receptors, it will synergize with signal 1 to further activate and expand those T cells that require both signals 1 and 2. CD28 agonism synergizes with signal 1 and even with 4-1BB agonism (32). Very strong CD28 agonism can even activate T cells on its own. This caused life-threatening CRS in the first-in-human trial of the CD28 superagonist TGN1412 and later led to the development of attenuated and targeted CD28 agonists (125).

There are two general approaches to deliver signal 2 with TCEs. The first is the parallel development of a companion bispecific antibody for combination with a CD3-targeted TCE (125, 126). Such a companion antibody is comprised of one arm which binds a target, such as fibroblast acidic protein (FAP), which is reliably expressed in the TME, and a second arm which agonizes a costimulatory receptor like CD28 or 4-1BB on TCE-engaged T cells (127). Clinical studies suggested that tumor anchoring can mitigate the known hepatotoxicity of 4-1BB agonism, however such a bispecific on its own only yields limited anti-tumor efficacy (127, 128). In preclinical mouse models, co-administration of a CEAxCD3 TCE with a FAPx4-1BB engager or of a CD20xCD3 TCE with a CD19x4-1BB engager had superior anti-tumor efficacy to single agents in mouse models (126). Likewise, CD22- or CD19-targeted CD28 agonists synergized with CD20-specific TCEs in mouse B cell lymphoma models (124). A combination of the CD20 TCE glofitamab with the CD19-targeted 4-1BB agonist englumafusp alfa elicited strong synergy and promising results in r/r B-NHL patients (124). Clinical data with such a combination approach in solid tumor indications was recently published, specifically the combination of the CEA-specific TCE cibisatamab with a FAP-targeted 4-1BB agonist. The combination had manageable safety with 3.8% DLTs in 52 patients, 73.1% grade 3+ and 65.4% serious adverse events, including 25% serious CRS and 7.7% grade 5 adverse events, although toxicities were lower after step-up dosing. Overall, the combination elicited an ORR of 13% which was higher than that of cibisatamab alone in other studies, although cross-study comparisons are to be taken with caution (46, 129). A caveat of this study was the inclusion of obinutuzumab to mitigate anti-drug-antibody formation against cibisatamab by depleting B cells.

The second approach to delivering both signals 1 and 2 to engaged T cells is to incorporate both a CD3 binder and a costimulatory agonist domain into the same, now tri-specific, TCE molecule (**Figures 5A, B**). Such constructs require a configuration that prevents fratricide, i.e., the lysis of T cells expressing the costimulatory receptor by those T cells engaged via CD3ϵ. This can be achieved, for example, via proximity or steric hindrance of the domains binding to CD3 and to the costimulatory receptor leading to mutually exclusive binding (130) or by employing a T cell-binding domain that is intrinsically dual-specific for CD3 and 4-1BB (33).

A fundamental question is whether providing signal 2 in the clinical setting, even within the same TCE molecule, will actually increase RRs and the duration of response. Furthermore, a potential concern is that costimulation could augment the frequency of adverse events from T cell activation via stimulation of autoimmune T cell clones in the naïve T cell repertoire. Several ongoing clinical trials may provide some initial answers (**Table 4**). The incidence of grade 3+ TRAEs (21.3%) including EBV or CMV reactivation and one grade 4 ICANS combined with low ORRs of 5% in rrMM and 14.3% in rrNHL for the CD3xCD38xCD28 TCE SAR442257 may explain why its phase 1 trial was terminated and indicate potential liabilities with this approach (131). Encouragingly, the CD3xCD19xCD28 TCE CC312 had a favorable safety profile without CRS or ICANS above grade 2 and with emerging efficacy in a few SLE patients (132).

**Table 4:** Costimulatory TCEs in clinical development.

Targets	Company	Asset	Indication(s)	Phase	Trial ID	References
CD3xCD38x**CD28**	Sanofi	SAR442257	MM, NHL	1	NCT04401020 (terminated)	(55, 131)
CD3xCD19x**CD28**	CytoCares	CC312	NHL, AID	1	NCT06037018, NCT06888960	(55, 132, 133)
CD19xCD3x**4-1BB**x**PD-L1**	Sichuan Baili Pharmaceutical Co	GNC-038	NHL, ALL, SLE, RA, DLBCL, PCNSL/SCNSL, TCL/NKCL	1/2	NCT04606433, NCT05623982, NCT06857214, NCT06857227, NCT05192486, NCT05485753, NCT05627856, NCT05189782	(55, 134)
CD3xCD19x**CD2**	Novartis	PIT565	NHL, ALL, RA, SLE	1	NCT05397496, NCT07029555, NCT06335979	(55, 135)
CD3xHER2x**CD28**	Sanofi	SAR443216	ST	1	NCT05013554	(55, 136)
DLL3xCD3x**4−1BB**	Chugai/Roche	Clesitamig (RO7616789/RG6524/ALPs12)	SCLC, neuroendocrine cancer	1	NCT05619744	(33, 55)
FAPx**4-1BB**xHSA	Molecular Partners	MP0310 (DARPin)	ST	1	NCT04049903	(55, 137)
PSMAx**4-1BB**xHSA	Crescendo Biologics	CB307	PSMA^+^ ST	1	NCT04839991	(55, 128)
CD3x**CD2**xULBP2/5/6	EvolveImmune Therapeutics	EVOLVE104	ST	1	NCT07217171.	(138)

Bold font indicates the costimulatory agonist or checkpoint blocking moiety. 4-1BB/CD137/TNFRSF9, tumor necrosis factor receptor superfamily member 9; AID, autoimmune disease(s); ALL, acute lymphoblastic leukemia; CD19, cluster of differentiation 19; CD2, cluster of differentiation 2; CD20, cluster of differentiation 20; CD28, cluster of differentiation 28; CD3, cluster of differentiation 3; CD38, cluster of differentiation 38; DLBCL, diffuse large B-cell lymphoma; DLL3, delta-like 3; FAP, fibroblast activation protein; HER2, human epidermal growth factor receptor 2; HSA, human serum albumin; MM, multiple myeloma; MSLN, mesothelin; NHL, Non-Hodgkin lymphoma; PCNSL, primary central nervous system lymphoma; PD-L1, programmed death-ligand 1; PSMA, prostate-specific membrane antigen; RA, rheumatoid arthritis; SCLC, small-cell lung cancer; SCNSL, secondary central nervous system lymphoma; SLE, systemic lupus erythematosus; ST, solid tumor; TCL, T-cell lymphoma; NKCL, natural killer cell lymphoma.

Additional early promise is emerging with incorporation of agonist-moieties for the co-stimulatory receptor CD2, which plays a critical signaling role in sustaining T cell fitness and overcoming exhaustion. CD2 on T cells can be agonized either by anti-CD2 antibodies or using the extracellular domain of CD58, the natural CD2 ligand. PIT565 is a trispecific TCE incorporating CD58 developed by Novartis. A preclinical study with this CD19-specific TCE suggested deeper and more durable responses (135). Phase 1 clinical trials with PIT565 are currently ongoing in SLE and RA patients (**Table 4**). Another clinical-stage TCE incorporating CD2 agonism is EVOLVE104, which targets the stress ligands ULBP2, 5 and 6 (**Table 4**) (138).

Thus, combining TCR engagement and costimulatory agonism within the same TCE molecule may be feasible, providing simplicity and convenience over co-administering two different agents. This is further supported by the clinical efficacy of the T cell subset-specific costimulatory TCE Invikafusp alfa discussed in the next section (139).

## TCEs engaging T cell subpopulations

By binding the invariant TCR subunit CD3ϵ, all commercial TCEs can theoretically engage the entirety of T cell phenotypes. This includes CD8, CD4, NKT and γδ T cells that are all potentially equipped with secretory granules containing perforin and granzymes to enable target cell lysis (140). The clinical experience demonstrates that pan-T cell engagement is feasible and can confer considerable clinical benefit despite CRS and ICANS as class toxicities of TCEs. A routine application of mitigation strategies has evolved that, when combined, can limit the initial CRS and ICANS to grade 2 (1). Nevertheless, numerous efforts are being made to develop TCEs that engage only a subset of T cells. One goal is to limit the release of inflammatory cytokines, the other is to avoid the activation of regulatory CD4+ T cells (Tregs), which are hypothesized to limit the overall activity of TCEs. Below, we discuss a few examples. Clinical assets are listed in **Table 5**.

**Table 5:** T cell subpopulation-targeting TCEs in clinical development .

Targets	Company	Asset	Indication(s)	Phase	Trial ID	References
Vβ6 or Vβ10 TCRxCD2	Marengo Therapeutics	Invikafusp alfa (STAR0602)	ST, BC	1/2	NCT05592626, NCT06827613	(55, 139, 141–144)
CD20xTCRxCD8	AstraZeneca	AZD5492	B cell malignancies, SLE, IIM, RA	1/2	NCT06542250, NCT06916806	(34, 55)
Vδ2 TCRxEGFR	Pfizer (Lava Therapeutics/Xoma Royalty)	PF-08046052 (LAVA-1223/​SGN-EGFRd2)	ST	1	NCT05983133	(55)
Vδ2 TCRxCD33	J&J (Lava Therapeutics/Xoma Royalty)	JNJ-89853413	AML/MDS	1	NCT06618001	(55, 145)
Vδ2 TCRxCD123	(Lava Therapeutics/Xoma Royalty)	LAVA-1266	AML/MDS	1	ACTRN12624001214527 (discontinued)	(146, 147)
Vδ2 TCRxPSMA		LAVA-1207	mCRPC	1	NCT05369000 (terminated)	(55, 148, 149)
Vδ2 TCRxCD1d		LAVA-051	CLL, MM, or AML	1	NCT04887259 (terminated)	(55, 150)
Anti-BRN3A mAb, activates Vγ9δ2 T cells	ImCheck Therapeutics	ICT01	AML/MDS, ALL, melanoma, ST	1/2	NCT04243499, NCT05307874	(151–155)

ALL, acute lymphoblastic leukemia; AML, acute myelogenous leukemia; BC, breast cancer; BRN3A, brain-specific homeobox/POU domain protein 3A; CD123, cluster of differentiation 123/interleukin-3 receptor; CD1d, cluster of differentiation 1d; CD2, cluster of differentiation 2; CD20, cluster of differentiation 20; CD33, cluster of differentiation 33/Siglec-3; CD8, cluster of differentiation 8; CLL, chronic lymphocytic leukemia; EGFR, epidermal growth factor receptor; IIM, idiopathic inflammatory myopathy; mCRPC, metastatic castration-resistant prostate cancer; MDS, myelodysplastic syndrome; MM, multiple myeloma; PSMA, prostate-specific membrane antigen; RA, rheumatoid arthritis; SLE, systemic lupus erythematosus; ST, solid tumors; TCR, T cell receptor.

One of the first attempts to selectively engage T cells by TCEs was by Yoram Reiter’s laboratory (132). They aimed to specifically engage T cells specific for the p65 antigen of cytomegalovirus (CMV). A majority of the human population has a high frequency of CMV-reactive effector memory T cells, the frequency of which increases with age. Instead of using an antibody fragment binding to CD3ϵ, they employed a soluble p65 peptide/MHC domain to target CMV-specific T cells, which they then fused to the target binding domain (156). Preclinical studies were successful, but such TCEs never reached the clinic. A limitation of this approach is its restriction to patients with HLA-A*02:01 and perhaps variable levels of CMV-reactive T cells.

Another approach is to only engage T cells expressing subsets of TCRs by targeting specific variable regions of the TCRα/β chains instead of the shared CD3ϵ subunit. In one example, Marengo Therapeutics’ STAR-platform comprises TCEs which selectively bind specific Vβ segments of the TCR beta chain. Invikafusp alfa (STAR0602) binds both Vβ6 and Vβ10 TCRs, present on ~10% of tumor-infiltrating T cells in certain cancers, and the costimulatory receptor CD2 on T cells. This TCE had a manageable safety with mostly low-grade adverse events including CRS and elicited meaningful single-agent efficacy in anti-PD(L)-1 resistant and -naïve solid tumors, including responses rates of 20.5-30% in tumors with a high mutation burden or high microsatellite instability/mismatch repair-deficiency (139, 141). A combination with the ADC sacituzumab govitecan in breast cancer patients was safe with signs of efficacy (142). It remains to be determined to what extent selective engagement of Vβ6/Vβ10 T cells versus pan-T cells impacts efficacy, and what costimulatory agonism may contribute in this setting.

Another approach is the development of TCEs that selectively engage the TCR of γδ T cells rather than the more abundant αβ T cells. Of note, conventional TCEs engage both populations of T cells by targeting the shared CD3ϵ subunit. While γδ T cells comprise less than 10% of T cells, the γδ TCR is not uniformly restricted to recognition of pMHC complexes but can also recognize non-peptide antigens, including lipids presented by CD1 and phosphorylated metabolites presented by MHC-like molecules. In one example, LAVA Therapeutics’ (now Xoma Royalty) gammabodies selectively bind the Vδ2 chain of Vγ9Vδ2 TCRs and engage Vγ9Vδ2 T cells. However, despite multiple clinical trials and reports of encouraging safety, clinical success thus far has been limited with several discontinuations (**Table 5**). Vγ9Vδ2 T cells are also selectively engaged by ICT01, ImCheck Therapeutics’ activating antibody against butyrophilin 3A (BTN3A/CD277) (151). ICT01 was safe without DLTs and had moderate efficacy in solid tumor patients in combination with pembrolizumab (15% ORR in melanoma) or IL-2 (152–154). In AML patients, ICT01/azacitidine-venetoclax was well tolerated and efficacious with 88% CR/CRi-rates (155). Taken together, these data suggest some benefit for single-agent Vγ9Vδ2 TCEs. Additional companies with γδ TCEs in preclinical development include IN8bio (157), Takeda/Adaptate Biotherapeutics [Vδ1 TCEs (158)], WuXi [pan-γδ TCEs (159)], and Tavotek, which develops costimulatory pan-γδ TCEs harboring IL-15R agonist fusions, including the CD123-targeting TAVO317A. Co-agonizing IL-15R aims at improving the so far limited clinical efficacy of γδ TCEs (160).

Mucosal-Associated Invariant T cells (MAIT) are a relatively abundant population of innate-like T cells, comprising up to 20% of total T cells in certain tissues (161). Their selective engagement by TCEs, as pursued by Biomunex, promises lower cytokine release than by CD8 and CD4 T cells and the avoidance of Treg cell activation (162). A bispecific targeting PD-L1 on tumors selectively engages MAIT cells by binding TCR Vα7.2 (163). A HER-specific MAIT-TCE is in preclinical development (162).

Finally, AstraZeneca has developed the so-called TITAN platform enabling TCEs to selectively engage CD8 T cells, which have the highest cytotoxic potential and produce lower levels of cytokines than CD4 T cells when engaged by TCEs. AZD5492 is a trispecific TCE in clinical development targeting both the TCR and CD8 on T cells and the B cell antigen CD20. It is in clinical trials for B cell malignancies and autoimmune diseases (**Table 5**; **Figure 5C**). In preclinical studies, AZD5492 preferentially engaged CD8 T cells and had potent anti-tumor efficacy similar to conventional TCEs but with significantly less systemic cytokine production, and was well tolerated in monkeys (34). Beyond AstraZeneca, iTabMed is also developing trispecific TCEs which bind CD3, CD8 and a TAA (164).

Pan-T cell engagement is now very well validated. It remains an open question whether engaging subpopulations of T cells will be on par with the efficacy of canonical TCEs or can significantly improve upon safety given a lower release of cytokines. The role of cytokines for the anti-tumor activity of TCEs should not be underestimated, and there is evidence that Treg cells can engage in TCE-mediated target cell lysis provided they express granzymes and perforin (140).

## Broadening the indication space for TCEs

TCEs are now well established for the treatment of late-stage cancers with a total of twelve approvals by FDA or EMA (1). Within oncology, there is, however, a need to expand TCE therapies into novel indications of high medical need. In hematological malignancies, TCEs have not yet been approved for the treatment of, for instance, acute and chronic myelogenous leukemia, myeloproliferative disease, or T cell lymphomas. For solid tumor therapy, the only approved TCEs are for the treatment of uveal melanoma, small cell lung cancer and malignant ascites from EpCAM-expressing cancers. Promising results are emerging from trials in late-stage prostate cancer patients with TCEs targeting PSMA, STEAP1 or KLK2 (3). However, there still is a scarcity of TCE targets to safely pursue indications such as breast, colorectal, liver, kidney, bladder, gastric, pancreatic, ovarian or non-small cell lung cancers. The application of conditional TCEs or targeting peptide/MHC has the potential to broaden the target space in oncology.

The majority of approved TCEs treat B cell malignancies by targeting CD19, CD20, BCMA or GPCR5D (1). Apart from treating ALL, NHL, FL and MM patients, the respective TCEs also effectively eliminate normal B cells, which can eventually be repopulated from stem cells in the bone marrow (12, 13). Various B cell-mediated autoimmune diseases, including multiple sclerosis, rheumatoid arthritis, vasculitis or pemphigus vulgaris, are being treated with approved mAbs or Fc-enhanced IgG1 mAbs targeting CD20, and in one case CD19. This prompted testing of CD19-, CD20- and BCMA-specific TCEs approved in oncology for their potential therapeutic applicability in various autoimmune diseases. CD19-specific blinatumomab and BCMA-specific teclistamab have shown remarkable clinical activity in early clinical studies in a variety of autoimmune diseases (2). The deep and lasting B cell depletion by these TCEs, often referred to as “immune reset”, appears to translate into profound disease modification and introduction of treatment-free periods. The emerging data also suggests that TCEs as a modality and/or specifically leveraging CD19 and BCMA as targets may significantly surpass the clinical activity of IgG1-based anti CD20 mAb therapies. A significant number of novel TCEs, the majority of which target BCMA and/or CD19, are in early clinical trials in a range of autoimmune diseases (2). In contrast to TCEs in oncology, in the context of autoimmune disease, there is increased emphasis on safety, convenience and potential outpatient use. This may require the design of novel TCEs, although the typically lower target cell load in autoimmune diseases versus cancer is thought to underly the observed reduced CRS and ICANS in autoimmune versus cancer patients (41, 165, 166) and may obviate the need for novel molecular designs. Efficacy can be optimized by repeat dosing and/or dose escalation, and safety may be addressed by adjusting dosing regimen, including step-up dosing, optimized selection of target dose levels and duration of dosing, and by subcutaneous administration (1).

The application of TCEs to an even broader indication space, beyond oncology and autoimmune diseases, holds early promise. Recently, a B cell-depleting TCE has been successfully used to prepare a transplant patient for allograft acceptance (167). Additionally, TCEs have potential in treating viral diseases. Preclinical proof-of-concept studies have shown that cells infected with SARS-CoV-2, influenza, HBV or CMV can be eliminated by TCEs recognizing respective viral proteins temporarily expressed or presented on the surface of infected cells (168–171). Moreover, viral protein-targeted TCEs encouragingly reduced levels of hepatitis B surface antigen in patients with chronic hepatitis B, and HIV viral load in AIDS patients (172). Finally, the approved BCMA TCE linvoseltamab is being explored for treating food allergies (173, 174). These examples highlight the opportunity for TCEs to extend into additional indication spaces in the future.

## Outlook

In recent years, TCEs have increasingly taken the stage in oncology as a novel modality demonstrating compelling single-agent activity. A dozen approved, first-generation TCEs are exhibiting impressive results as stand-alone therapy, in minimal residual disease (MRD) settings or in combination with standard of care (SoC). A wide range of SoCs can augment the efficacy of first-generation TCEs and vice versa, including chemotherapies, ADCs, mAbs, imids, tyrosine kinase inhibitors and checkpoint inhibitors. Such combinations may enable TCEs to move into first-line settings and may allow sparing chemotherapeutics. There are still areas for potential improvement of TCE design and intrinsic features, with the promise to enhance efficacy with the addition of costimulatory domains, reduce toxicities by targeting subsets of T cells or through conditional design, address heterogenous target expression with logic gating, and thereby broaden the impact of TCEs,. As a targeted therapy approach, it will also be important to identify novel target antigens allowing this modality to move into an even number of cancer indications. The advance of TCEs into indications beyond oncology is another burgeoning frontier, with initial data suggesting promising efficacy in a range of autoimmune diseases and additional promise to extend into an increasingly expansive indication space.
